# Can Quantitative Pupillometry be used to Screen for Elevated Intracranial Pressure? A Retrospective Cohort Study

**DOI:** 10.1007/s12028-022-01518-y

**Published:** 2022-05-23

**Authors:** Jakob Pansell, Robert Hack, Peter Rudberg, Max Bell, Charith Cooray

**Affiliations:** 1 Department of Clinical Neuroscience, Karolinska Institutet, Central Intensive Care Unit E5:67, Karolinska University Hospital, Stockholm, 17176 Sweden; 2 Department of Anesthesia and Intensive Care Medicine, Karolinska Institutet, Central Intensive Care Unit E5:67, Karolinska University Hospital, Stockholm, 17176 Sweden; 3 Department of Physiology & Pharmacology, Karolinska Institutet, Stockholm, Sweden; 4 The Department of Clinical Neurophysiology, Karolinska University Hospital, Stockholm, Sweden

**Keywords:** Intracranial pressure, Quantitative pupillometry, Brain injuries, Neurology, Neurosurgery, Critical care, Stroke, Cardiac arrest, Brain edema, Cerebral edema, Intensive care

## Abstract

**Background:**

Elevated intracranial pressure (ICP) is a serious complication in brain injury. Because of the risks involved, ICP is not monitored in all patients at risk. Noninvasive screening tools to identify patients with elevated ICP are needed. Anisocoria, abnormal pupillary size, and abnormal pupillary light reflex are signs of high ICP, but manual pupillometry is arbitrary and subject to interrater variability. We have evaluated quantitative pupillometry as a screening tool for elevated ICP.

**Methods:**

We performed a retrospective observational study of the association between Neurological Pupil index (NPi), measured with the Neuroptics NPi-200 pupillometer, and ICP in patients routinely monitored with invasive ICP measurement in the intensive care unit. We performed a nonparametric receiver operator curve analysis for ICP ≥ 20 mm Hg with NPi as a classification variable. We performed a Youden analysis for the optimal NPi cutoff value and recorded sensitivity and specificity for this cutoff value. We also performed a logistic regression with elevated ICP as the dependent variable and NPi as the independent variable.

**Results:**

We included 65 patients with invasive ICP monitoring. A total of 2,705 measurements were analyzed. Using NPi as a screening tool for elevated ICP yielded an area under receiver operator curve of 0.72. The optimal mean NPi cutoff value to rule out elevated ICP was ≥ 3.9. The probability of elevated ICP decreased with increasing NPi, with an odds ratio of 0.55 (0.50, 0.61).

**Conclusions:**

Screening with NPi may inform high stakes clinical decisions by ruling out elevated ICP with a high degree of certainty. It may also aid in estimating probabilities of elevated ICP. This can help to weigh the risks of initiating invasive ICP monitoring against the risks of not doing so. Because of its ease of use and excellent interrater reliability, we suggest further studies of NPi as a screening tool for elevated ICP.

## Introduction

Elevated intracranial pressure (ICP) is a serious complication in brain injury. It can impede blood flow through the brain and lead to secondary brain injury, resulting in poor neurological outcome and even death [1]. Aggressive treatment of elevated ICP is a cornerstone of neurointensive care, but to treat elevated ICP adequately and safely, it needs to be monitored. ICP monitoring requires neurosurgical procedures and are associated with risks such as bleeding and infection. In Sweden, it also necessitates transfer to a neurointensive care unit. Many patients at risk are therefore not monitored with respect to ICP [2]. Noninvasive screening tools to identify patients truly at risk of elevated ICP have long been sought after. Patients who could benefit from such monitoring include survivors of cardiac arrest or stroke, patients with hepatic encephalopathy, or patients with central nervous system infections [2].

Several noninvasive surrogate parameters for increased ICP have been studied. The most promising of these are transcranial Doppler sonography and measurement of the optic nerve sheath diameter. Both methods require trained and experienced operators [2–5]. Anisocoria, abnormal pupillary size, and abnormal pupillary light reflex are well-known signs of high ICP. This is mainly thought to occur as a result of mechanical compression, hypoperfusion, or hypoxia of the oculomotor nerve or the optic nerve [1, 6, 7]. Manual pupillometry performed with a penlight has shown substantial interexaminer variability, very low consistency, and relies on subjective and imprecise descriptions [6, 8]. Quantitative pupillometry has been developed to remedy these shortcomings: the method is quantitative, reproducible, and has shown excellent interexaminer reliability [6, 8, 9]. It presents precise values of pupillary size and latency, as well as velocity and percentage of pupillary constriction when the eye is exposed to light. In addition, the Neuroptics NPi-200 pupillometer presents a composite value termed Neurological Pupil index (NPi). NPi is calculated from pupillary size, latency of reaction, velocity of constriction and dilation, and percentage of change. NPi is suggested to be minimally influenced by pharmacological effects [8]. NPi has an inverse relationship to ICP [6, 8], but the association is weak [10, 11]. Anisocoria has shown an association with elevated ICP, and in a case series of three patients there was a significant side difference in NPi during deterioration due to herniation [12]. Minimum NPi also has shown an association with midline shift [13]. Beyond that, side difference in NPi has, to our knowledge, not been studied for an association with ICP [1, 6], nor has NPi been extensively studied for an association with cerebral perfusion pressure (CPP). To date, NPi is not an established ICP estimate or surrogate. However, it can be easily performed by unskilled personnel, it is operator independent, and it is widely accessible in most care settings. All of these are qualities sought for in tools for noninvasive ICP estimation. We have performed a retrospective cohort study to evaluate mean NPi, minimum NPi, and NPi side difference as screening tools for the prediction of elevated ICP and low CPP.

## Methods

### Participants

The setting for this study was the general intensive care unit (ICU) and the neurointensive care unit at Karolinska University Hospital in Stockholm, Sweden. The units have a combined capacity of 16 ICU beds with several hundred patients undergoing invasive ICP monitoring every year. We included all patients included in an ongoing study on noninvasive ICP estimation using optic nerve sheath diameter and transcranial Doppler sonography. Inclusion criteria for this study were adult patients treated in the ICU, sedated or unconscious, and monitored with invasive ICP measurement as standard of care. Exclusion criteria were ocular disease or ocular trauma. Quantitative pupillometry is routinely performed in these patients by intensive care nurses at least three times daily. The nature of this cohort makes informed consent unfeasible. Next of kin were informed and given the right to opt out on behalf of the patient. The study was conducted in accordance with the Helsinki declaration and was approved by the Swedish Ethical Review Authority, record number 2020–03,004.

### Data Extraction

Data were manually extracted from electronic medical records. ICP and CPP values are automatically registered in the electronic medical records, normally with 2-min intervals, but occasionally longer intervals because of technicalities. NPi is performed routinely by ICU nurses at least three times daily and at the individual clinician´s discretion with shorter intervals. NPi values are manually recorded in the electronic medical records. When ICP and NPi were not recorded the same minute, we averaged the ICP values before and after NPi registration, if not separated from the NPi value by more than 5 min. All measurements from the full range of possible NPi values (0 to 5) were included in the analysis.

### Statistical Analysis

All patients contributed different amounts of data. We therefore performed linear regression analyses adjusted for clusters, with every patient defined as one cluster. This statistical model applies robust standard errors computed on the basis of aggregates from the clusters. Mean NPi, minimum NPi, and NPi side difference were independent variables in the different linear regression analyses. ICP or CPP were dependent variables. Mean NPi was defined as the mean of left and right NPi at the time of measurement. NPi side difference was defined as the difference between left and right NPi in absolute numbers at the time of measurement.

We performed a receiver operator characteristic (ROC) analysis for elevated ICP with NPi as classification variable, on the basis of a logistic regression analysis with robust standard errors to compensate for the different amounts of data contributed by each patient. ICP measurements ≥ 20 mm Hg were classified as elevated and measurements < 20 mm Hg were classified as normal, in line with most previous studies of quantitative pupillometry to estimate ICP [6]. We performed a Youden analysis on the basis of the same logistic regression analysis with robust standard errors for the optimal NPi cutoff value to identify elevated ICP and recorded sensitivity and specificity for this cutoff value. We also calculated positive predictive values (PPV) and negative predictive values (NPV) for the cutoff value. Analyses were performed on the whole cohort and stratified by diagnosis and by sex. All analyses were performed in Stata version 14.2.

## Results

We included 65 patients (29 women, 36 women) with a median age of 54 years (interquartile range 42 to 63 years). Patients contributed to a total of 2705 measurements with ICP and bilateral NPi values. Most common diagnoses were subarachnoid hemorrhage (SAH), intracranial hematoma, and traumatic brain injury (see Table 1). Five patients had diabetes, and these patients had a lower average NPi (3.7 vs. 3.9, *p* = 0.01) and a lower average ICP (10.0 vs. 11.6, *p* = 0.001) compared with the other patients. We removed 12 measurements (0.4%) with obvious data entry errors in the patient charts (recorded NPi values off the NPi scale). Because of the very small number, these measurements could be removed without risk of introducing bias. Two patients (3%) had missing data for comorbidity. These were not removed from the data set because comorbidity was not part of our main analysis and there was no risk of introducing bias by keeping these. The final data set forming the base for the current analysis consisted of 2,693 measurements. Of these measurements, 2,619 contained CPP data (see Table 1 for descriptive data of the cohort). The diagnosis of SAH was more common in women, and traumatic brain injury was more common in men. There were also slight differences between men and women with respect to mean ICP and mean NPi (see Table 2).

**Table 1:** Descriptive data of the cohort

Characteristic	Patients, *N* = 65
Female sex, *n*/*N* (%)	29/65 (44.6)
Age, median (interquartile range) (yr)	54 (42–63)
Number of measurements per patient, median (interquartile range)	33 (15–55)
Primary diagnosis, *n*/*N* (%)	
Subarachnoid hemorrhage	26/65 (40.0)
Traumatic brain injury	19/65 (29.2)
Intracerebral hematoma	10/65 (15.4)
Other primary diagnosis	10/65 (15.4)
Comorbidities, *n*/*N* (%)	
Cardiovascular disease	7/63 (11.1)
Asthma/chronic obstructive pulmonary disease	5/63 (7.9)
Diabetes mellitus	5/63 (7.9)
Treatment, *n*/*N* (%)	
Invasive ventilation	65/65 (100)
Propofol	59/65 (90.8)
Midazolam	23/65 (35.4)
Pentothal	5/65 (7.9)
Opiates	52/65 (80.0)
Vasopressors	51/65 (78.5)
Inotropes	7/65 (10.8)

**Table 2:** Sex differences in the cohort

Characteristic	Male sex	Female sex	*p* value
Subarachnoid hemorrhage, *n* (%)	10 (27.8)	16 (55.2)	0.02
Intracerebral hematoma, *n* (%)	5 (13.9)	5 (17.2)	0.49
Traumatic brain injury, *n* (%)	15 (41.7)	4 (13.8)	0.01
Other diagnosis, *n* (%)	6 (16.7)	4 (13.8)	0.51
NPi, mean (95% CI)	3.80 (3.75–3.85)	4.00 (3.95–4.05)	0.00
ICP, mean (95% CI)	12.3 (11.9–12.8)	10.6 (10.1–11.1)	0.00
Occurrence of elevated ICP, % (95% CI)	7.1 (5.8–8.4)	6.8 (5.3–8.2)	0.70

CI, confidence interval, ICP, intracranial pressure, NPi, Neurological Pupil index

Linear regression yielded a significant negative correlation (*β* =  − 2.8, *p* = 0.000) between mean NPi and ICP but with a poor fit (*r*^2^ = 0.083). Linear regression yielded a significant negative correlation (β =  − 2.1, *p* = 0.000) between minimum NPi and ICP but with a poor fit (*r*^2^ = 0.07). Linear regression yielded a significant positive correlation (*β* = 1.3, *p* = 0.000) between NPi side difference and ICP but with a very poor fit (*r*^2^ = 0.009). All linear regressions were adjusted for intraperson correlation.

Linear regression yielded a statistically significant correlation (*β* = 1.8, *p* = 0.000) between mean NPi and CPP but with a very poor fit (*r*^2^ = 0.008). Linear regression yielded a statistically significant correlation (*β* = 1.5, *p* = 0.000) between minimum NPi and CPP but with a very poor fit (*r*^2^ = 0.009). Linear regression yielded a statistically significant negative correlation (*β* =  − 1.8, *p* = 0.000) between NPi side difference and CPP but with a very poor fit (*r*^2^ = 0.004). All linear regressions were adjusted for intraperson correlation. Because of the virtually nonexistent associations between ICP and NPi side difference, CPP and mean NPi, CPP and minimum NPi, and CPP and NPi side difference, we did not analyze diagnostic or predictive performance for any of these.

**Fig. 1:**
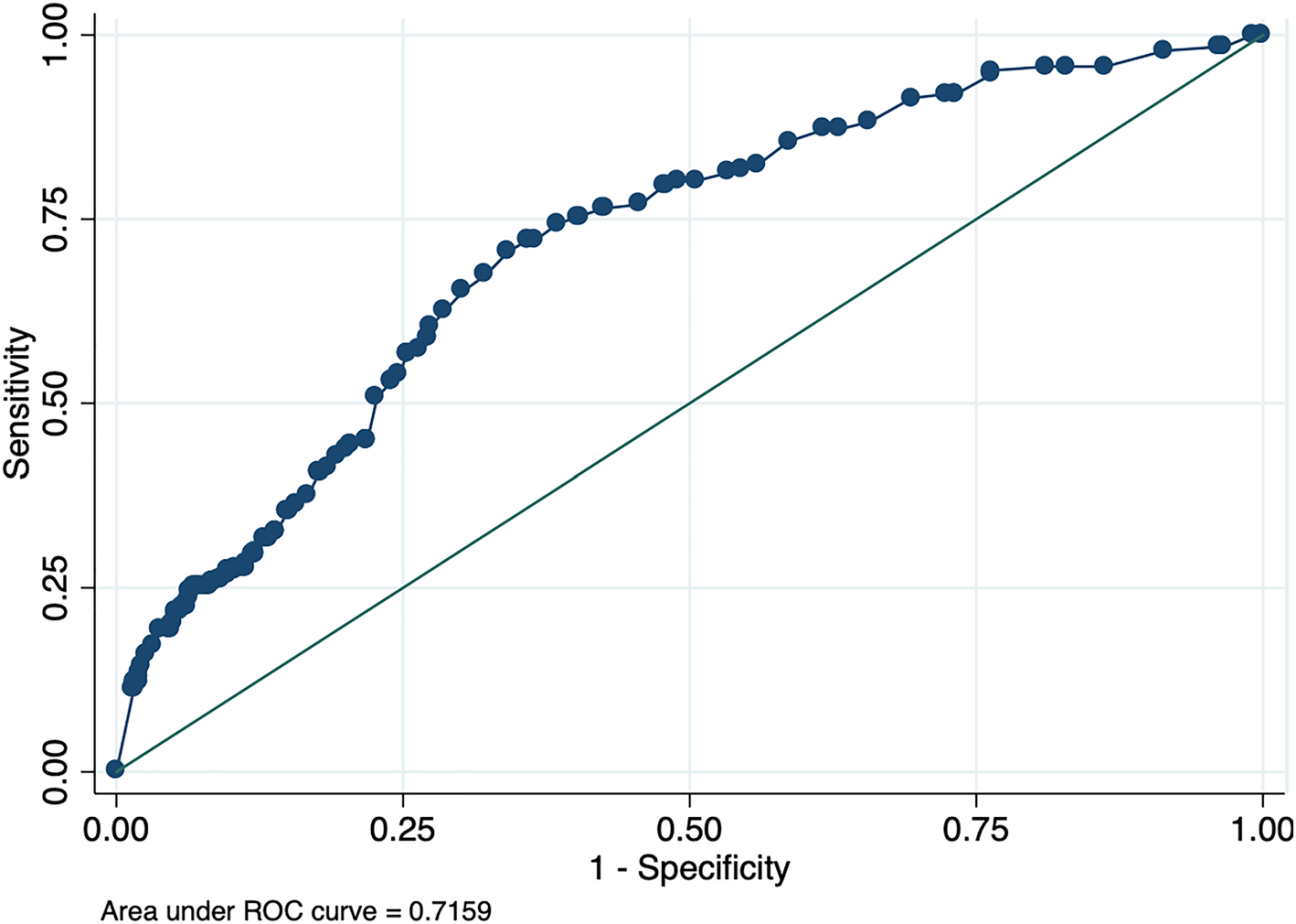
Area under receiver operator characteristic (ROC) curve for mean Neurological Pupil index in predicting elevated intracranial pressure

**Fig. 2:**
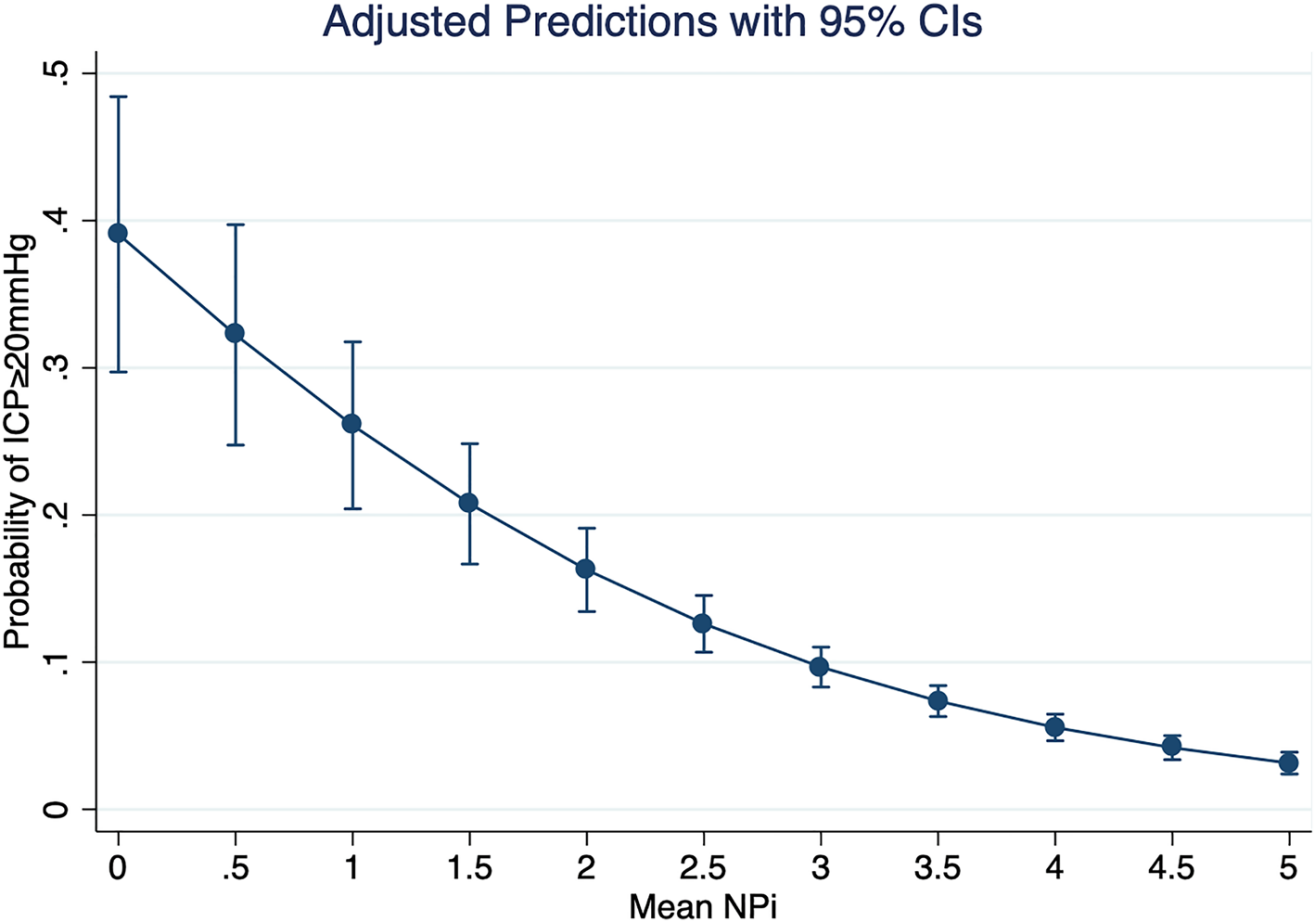
Predicted probability of elevated intracranial pressure decreases with increasing mean Neurological Pupil index (NPi). Odds ratio is 0.55 (0.50, 0.61). CI, confidence interval

**Fig. 3:**
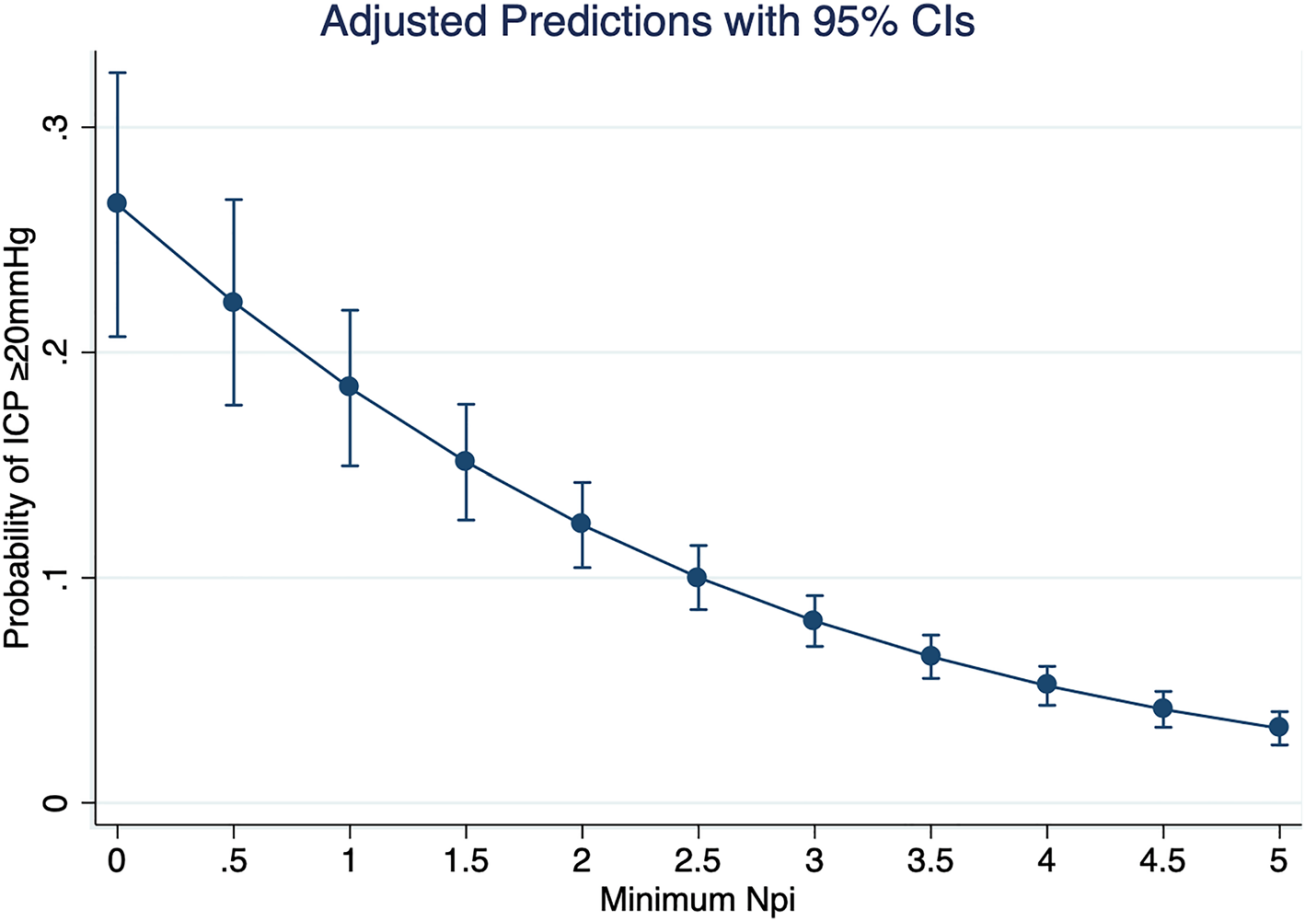
Predicted probability of elevated intracranial pressure decreases with increasing minimum Neurological Pupil index (NPi). Odds ratio is 0.62 (0.57, 0.68). CI, confidence interval

ICP was elevated (≥ 20 mm Hg) in 188 measurements (7%). These events occurred in 23 patients (35%). ROC analysis for elevated ICP with mean NPi as classification variable yielded an area under the ROC curve (AUROC) of 0.72. The Youden analysis generated an optimal mean NPi cutoff at 3.85 (see Fig. 1). ROC analysis for elevated ICP with minimum NPi as classification variable yielded an AUROC of 0.71. The Youden analysis generated an optimal cutoff for minimum NPi at 3.70. The difference in AUROC between mean NPi and minimum NPi was not statistically significant. Table 3 shows AUROC, optimal cutoff, sensitivity, specificity, PPV, and NPV for the cohort as a whole and for different strata. Logistic regression with elevated ICP as the dependent variable and mean NPi as the independent variable yielded a decrease in predicted probability of elevated ICP with increasing NPi, with an odds ratio of 0.55 (0.50, 0.61, see Fig. 2). Logistic regression with elevated ICP as the dependent variable and minimum NPi as the independent variable yielded a decrease in predicted probability of elevated ICP with increasing NPi, with an odds ratio of 0.62 (0.57, 0.68, see Fig. 3).

**Table 3:** Stratified analysis of Neurological Pupil index to identify elevated intracranial pressure

Characteristic	AUROC	Youden cutoff	Sensitivity	Specificity	Positive predictive value (%)	Negative predictive value (%)
All patients	0.72	3.85	0.70	0.66	13.4	96.7
Subarachnoid hemorrhage	0.73	3.775	0.65	0.75	9.6	98.2
Intracerebral hematoma	0.71	3.975	0.86	0.58	20.4	97.1
Traumatic brain injury	0.63	3.775	0.66	0.60	16.9	92.4
Others	0.78	3.075	0.62	0.90	12.2	99.1
Male sex	0.69	3.925	0.70	0.61	12.2	96.4
Female sex	0.75	3.975	0.78	0.66	14.1	97.6

AUROC, area under receiver operator characteristic curve

## Discussion

This study shows that mean NPi and minimum NPi may provide valuable clinical information in screening for elevated ICP but not for low CPP. It seems to be particularly useful as a “rule out” tool with high NPV at a suggested cutoff rounded to ≥ 3.9 for mean NPi and ≥ 3.7 for minimum NPi. It may also be feasible to use NPi to predict probabilities of elevated ICP across the spectrum of NPi values. Mean NPi performed slightly but not significantly better than minimum NPi in predicting elevated ICP. NPi side difference does not seem to add any information of value in screening for elevated ICP.

Regarding the number of patients, this is one of the largest studies investigating the association between NPi and ICP. It is also one of the first studies investigating prediction of probability of elevated ICP as a function of mean NPi or minimum NPi. It is one of the first studies investigating the association between NPi and CPP and, to the best of our knowledge, the first study investigating the associations between NPi side difference and ICP or CPP.

The results from this study are similar to results from previous studies regarding cutoff, sensitivity, specificity, and AUROC for ICP prediction using NPi [10, 11, 14]. The negative association between NPi and ICP, however, was somewhat weaker in our study than in the previous studies. Our cohort was comparable with two of these previous studies with a mixed sample of primarily SAH, traumatic brain injury, and intracranial hematoma [10, 11]. Notably, the optimal mean NPi cutoff to identify elevated ICP in our study was higher than the previously published threshold of < 3 for pathological NPi [6]. This is in line with recent findings by Robba et al. [10].

In the current study, the relationship between NPi and ICP has been assumed to be instantaneous, with a direct correlation between NPi and ICP at a given time point.

In clinical practice, however, there is often a time lag of a few minutes between the actual measurement of NPi and the manual recording of NPi in the patient charts. In this retrospective study, we only had access to the time of manual recording of NPi, not the actual time of measurement. When comparing NPi measurements with ICP, there may therefore exist a potential difference in timing for the two measurements. If large changes in ICP did occur between the time of NPi measurement and NPi recording, however, this should be considered at random and not in a systematic way that affects the results of this study. Another limitation with this study is the assumption that recorded ICP equals the true ICP. Measurement errors may occur for a number of reasons that we were unable to detect because of the retrospective nature of this study. Likewise, the 12 entries with NPi off the scale shows that data entry errors occurred in our data set. Because these are retrospective data, we have no way to evaluate the extent of data entry errors consisting of erroneous NPi values that are within the NPi scale. This is a limitation.

Delayed cerebral ischemia (DCI) in SAH was preceded by a decrease in NPi in a large proportion of patients in one study [15]. With 40% of patients having SAH in our cohort, we cannot rule out a possible effect of DCI on NPi. Because AUROC was very similar in the SAH stratum compared with the whole cohort (see Table 3) we do not believe this to be a major concern.

Pupillary reflexes may also be affected by other phenomena than elevated ICP or DCI. Several pathologic conditions affecting the optic nerve or the oculomotor nerve may affect NPi in the absence of elevated ICP [6] and may have occurred in patients in our cohort. With limited resources, we were not able to search patient charts for other pathologic conditions, possibly affecting pupillary reflexes at the time of NPi recordings. NPi also is affected by ambient light, especially in critically ill patients [16]. We could not control for ambient light in this retrospective study design, which may have diluted the correlation between ICP and NPi. For future prospective studies, we recommend standardized ambient light when performing NPi measurements. It has long been known that pupillary light reflexes can be impaired in diabetes [17]. In this study, average NPi was significantly lower in the patients with diabetes, despite a lower average ICP. There were only five patients with diabetes in our study, so this should be interpreted with caution. Still, it raises the question whether NPi is less reliable for ICP screening in patients with diabetes.

Although this study is relatively large compared with previous studies, our sample size of only 65 patients must be regarded as a limitation. The predictive probabilities of elevated ICP as a function of NPi as well as PPV and NPV are dependent on our base rate of 7% elevated ICP. A low PPV and a high NPV are to be expected with our pretest probability of 7% but will be different in a population with a different proportion of patients with elevated ICP. The relatively small proportion of elevated ICP and of low NPi are reflected in the wide confidence intervals for predictive probabilities with lower NPi values. The predictive probabilities and PPV and NPV at the suggested cutoff should be interpreted with caution. They need to be validated in a different, preferably larger, cohort to draw conclusions on the generalizability of our findings. Despite these limitations, our study is relatively large with high-quality data. The proportion of missing or erroneous data of interest to the analysis was negligible at 0.4%, and these data were removed without the risk of systematically affecting results. The missing data on comorbidities in two patients did not affect results because comorbidities were not included in the analysis.

## Conclusions

Mean NPi and minimum NPi may be of value as screening tools for elevated ICP. In particular, they show promising NPVs. In many settings, knowledge of the patient’s cerebral condition is sparse. Still, a decision needs to be made on whether the patient should be referred to a neurointensive care center for surgical intervention and/or invasive ICP monitoring. NPi may inform these high-stake clinical decisions by predicting probabilities of normal or elevated ICP.
